# Higher body mass index associated with severe early childhood caries

**DOI:** 10.1186/s12887-016-0679-6

**Published:** 2016-08-20

**Authors:** Katherine Davidson, Robert J. Schroth, Jeremy A. Levi, Aaron B. Yaffe, Betty-Anne Mittermuller, Elizabeth A. C. Sellers

**Affiliations:** 1 The University of Manitoba, Winnipeg, Canada; 2 The Children’s Hospital Research Institute of Manitoba, Winnipeg, Canada; 3 Winnipeg Regional Health Authority, Winnipeg, Canada; 4 Department of Preventive Dental Science, Rady Faculty of Health Sciences, College of Dentistry, University of Manitoba, 507-715 McDermot Avenue, Winnipeg, MB R3E 3P4 Canada; 5 Department of Pediatrics & Child Health, Max Rady College of Medicine, University of Manitoba, 507-715 McDermot Avenue, Winnipeg, MB R3E 3P4 Canada

**Keywords:** Early childhood caries, Body mass index, Preschool, Child

## Abstract

**Background:**

Severe Early Childhood Caries (S-ECC) is an aggressive form of tooth decay in preschool children affecting quality of life and nutritional status. The purpose was to determine whether there is an association between Body Mass Index (BMI) and S-ECC.

**Methods:**

Children with S-ECC were recruited on the day of their slated dental surgery under general anesthesia. Age-matched, caries-free controls were recruited from the community. All children were participating in a larger study on nutrition and S-ECC. Analysis was restricted to children ≥ 24 months of age. Parents completed a questionnaire and heights and weights were recorded. BMI scores and age and gender adjusted BMI z-scores and percentiles were calculated. A *p*-value ≤ 0.05 was significant.

**Results:**

Two hundred thirty-five children were included (141 with S-ECC and 94 caries-free). The mean age was 43.3 ± 12.8 months and 50.2 % were male. Overall, 34.4 % of participants were overweight or obese. Significantly more children with S-ECC were classified as overweight or obese when compared to caries-free children (*p* = 0.038) and had significantly higher mean BMI z-scores than caries-free children (0.78 ± 1.26 vs. 0.22 ± 1.36, *p* = 0.002). Those with S-ECC also had significantly higher BMI percentiles (69.0 % ± 29.2 vs. 56.8 % ± 31.7, *p* = 0.003). Multiple linear regression analyses revealed that BMI z-scores were significantly and independently associated with S-ECC and annual household income as were BMI percentiles.

**Conclusions:**

Children with S-ECC in our sample had significantly higher BMI z-scores than caries-free peers.

## Background

Early childhood oral health plays a significant role in the overall health and well-being of young children [1]. Healthy teeth allow for optimal function in eating and speaking and provide an acceptable appearance facilitating early socialization. Good oral health and hygiene practices during early life sets the foundation for continued optimal oral health throughout childhood and into adolescence and adulthood [2].

While early childhood caries (ECC) is a very broad case definition that includes children with one or more primary teeth affected by decay, many children suffer from a more severe form of ECC, termed Severe Early Childhood Caries (S-ECC) [3]. The case definition for S-ECC is based on the child’s age and the location and severity of decay [3]. At-risk populations for S-ECC include those from socioeconomically deprived communities, Indigenous children, and recent newcomers to Canada [1, 4–6]. Children with S-ECC routinely undergo dental rehabilitative surgery under general anesthesia (GA). The implications of severe decay extend beyond the oral cavity and have been linked with poor nutrition and health status [1, 7–10]. Children with S-ECC may suffer from pain and infection that can lead to altered eating and sleeping habits, altered growth (specifically in weight and height), and altered behaviour [1, 11–16]. They are also at increased risk for future dental disease throughout life [1, 17].

Recently, there has been considerable interest in the relationship between childhood caries and childhood growth. Body mass index (BMI), expressed in kg/m^2^, is a measure of “body fatness” and is used in children ≥ 24 months of age [18]. BMI is age and gender specific, and can be expressed as a z-score allowing for comparisons between ages and genders. Current evidence suggests that severe caries is associated with malnutrition, which may be expressed as deviation from average BMI values [1, 14, 16, 19]. BMI is an effective screening tool, however it has comparative limitations due to inherent male/female differences in body fat percentage [18]. Fortunately, the Center for Disease Control (CDC) has generated BMI-for-age growth curves to separately assess males and females in children and youth [20]. These curves stand to overcome the limitation in using BMI to compare individuals of differing ages and sexes, by generating a percentile ranking from a z-score based on norms for children of the same age and sex so that they can be reliably compared with others [18].

BMI is classified into four categories; underweight, healthy, overweight and obese [18]. These categories correspond with specific score ranges in adults and specific percentile ranges in children. Both ends of the spectrum can be problematic with respect to health [16, 18]. Underweight may be a sign of malnutrition, which can leave a child at risk for altered growth, being immunocompromised, and developing other morbidities and micronutrient deficiencies [21]. Overweight and obese individuals are at increased risk for type 2 diabetes, hypertension and cardiovascular disease along with other chronic diseases [22].

Several studies have attempted to evaluate the association between BMI and S-ECC, with varied and sometimes conflicting results [14, 16, 19, 23, 24]. The earliest studies on this topic, as well as a very recent publication, have linked S-ECC with underweight and failure-to-thrive, suggesting that low BMI’s may be a consequence of S-ECC [13, 16, 25, 26]. Meanwhile, other studies have found significant associations between ECC and overweight, drawing conclusions that the connection may be based upon shared risk factors of the two outcomes [27, 28]. To further complicate the matter, additional population-based studies have found no significant associations between the two variables [23, 24, 29]. A recent review of the impact of S-ECC on early childhood health and well-being suggested that the relationship between extensive dental decay and childhood growth and development is not entirely clear, but may contribute to low weight. [1] To date, there is no published Canadian data on BMI and severe caries in young children.

The purpose of this study was to evaluate whether a significant association between BMI and S-ECC exists in Canadian preschool children enrolled into a larger study investigating the relationship between S-ECC and childhood nutritional status.

## Methods

Approval to conduct this research was granted by the Health Research Ethics Board at the University of Manitoba, the Misericordia Health Centre (MHC), and the Health Sciences Centre prior to the initiation of data collection. The project’s objectives and methodology were explained to parents and caregivers and written informed consent was obtained. Participants were predominantly residents of southern Manitoba and were recruited between the months of October 2009 and August 2011. The study adhered to the STROBE guidelines.

Participants in this sub-study were part of a larger research project examining the association between S-ECC and childhood nutritional status [9, 10]. The parent study assessed whether there were differences in vitamin D (25(OH)D), calcium, and ferritin levels between children with S-ECC and cavity-free controls. The sample size calculation for the parent study was based on vitamin D concentrations as the primary outcome of interest [10]. The two previous publications reported that children with S-ECC are significantly more likely to have iron deficiency anemia, and low levels of ferritin, vitamin D (25(OH)D), calcium, and albumin [9, 10]. A cross-sectional case-control study was undertaken to test the hypothesis that BMI differs significantly between children with S-ECC and caries-free controls. Measured heights and weights were obtained from participants, while each parent or primary caregiver completed an interviewed questionnaire administered by a member of the research team. The questionnaire asked a series of questions pertaining to each child’s nutritional habits, use of supplements, physical and oral health, oral hygiene and dental habits, socioeconomic status (e.g. household income), and family demographics.

S-ECC was defined in accordance with the American Academy of Pediatric Dentistry’s (AAPD) case definition for ECC and S-ECC [3]. Since both ECC and S-ECC are age specific definitions, participation was restricted to those < 72 months of age. All children undergoing dental surgery under GA had rampant tooth decay fulfilling the criteria for S-ECC. Our recruitment guidelines specified that children undergoing dental surgery to deal with dental injuries and minor oral surgery unrelated to caries were ineligible to participate. The majority of children with S-ECC were recruited on the day of their scheduled dental surgery at the MHC. Cavity-free (dmft = 0) preschool children serving as controls were recruited from the community and underwent a dental screening assessment by a licensed dentist to confirm that they were caries-free and following World Health Organization methodology for oral health surveys [30].

BMI was calculated by entering the recorded weight and height of the subjects into the CDC on-line Child and Teen BMI Percentile Calculator [31]. Pre-admission heights and weights for those in the surgery group were obtained from the hospital record. These measurements were made by trained and experienced healthcare professional on a calibrated digital Detecto Pro-Doc scale and stadiometer on the day of surgery. Those recruited in the community were measured by a trained member of the research team using a calibrated Detecto weigh beam scale and stadiometer. For the purpose of this study, we restricted eligibility to children between 24 and 71 months of age, as BMI measurement is not used in children < 24 months. BMI was converted to BMI z-scores and percentile data using an on-line calculator [32] in order to appropriately compare subjects of differing ages and genders. BMI percentiles were then classified into one of four categories, as per the CDC: underweight <5^th^ percentile, normal weight 5^th^ to < 85^th^ percentile, overweight 85^th^ to < 95^th^ percentile, and obese ≥ 95^th^ percentile [18].

Venipunctures for children with S-ECC were drawn by the attending anesthetist during surgery while blood samples from controls were obtained by a research nurse at the Children’s Hospital Research Institute of Manitoba following the application of a topical anaesthetic (EMLA) to the anticubital fossa. Assays for 25(OH)D, the main circulating form of vitamin D, were conducted by the Hospitals in Common Laboratory at Mount Sinai Hospital in Toronto, Canada using Chemiluminescence Immunoassay. 25(OH)D levels ≥ 75 nmol/L were considered optimal [10, 33, 34].

Data were compiled and entered into an Excel database (Microsoft Office) and analyzed using Number Cruncher Statistical Software (NCSS, Kaysville, Utah). Descriptive analyses included frequencies and means (standard deviations (SD)). Bivariate tests included Chi-square analysis and T-tests. Normally distributed data was analyzed with an Equal-Variance T-Test, while any data not normally distributed was assessed with the Aspin-Welch Unequal-Variance Test. Because BMI z-scores and BMI percentiles are measured on a continuous scale, each was modeled using multiple linear regression. The independent variables considered for each model were those found to be associated the dependent variable in bivariate analysis. A *p*-value ≤ 0.05 was significant.

## Results

Overall 266 children were recruited into the parent study [9, 10]. Of these, 241 children were between 24 and 71 months of age. Six children were excluded because of missing height or weight measurements. Of these 235, 141 had S-ECC and 94 were caries-free. The mean age was 43.3 (12.8) months and 118 (50.2 %) of the participants were male (Table 1). The S-ECC and caries-free groups did not differ significantly for age (*p* = 0.09) or sex (*p* = 0.46). Children with S-ECC were significantly more likely to belong to households with low annual incomes (< $28,000) (*p* < 0.001). Parents and caregivers of children with S-ECC were significantly less likely to have pursued post-secondary education (*p* < 0.001). According to parental and caregiver ratings, children with S-ECC also had poorer overall health ratings than caries-free children (*p* < 0.001) (Table 1). Caregivers of children with S-ECC were also significantly more likely to be recipients of government aid (e.g. social assistance) than those of caries-free children (57.1 % vs. 18.5 %, *p* < 0.001). However, there were no differences between the groups with respect to the frequency of snacking (*p* = 0.21).

**Table 1:** Participant characteristics and associations with S-ECC

Variable	Totals (*n* = 235)	S-ECC (*n* = 141)	Caries-Free (*n* = 94)	*P*-value
Sex				0.46^a^
Male	118	68 (57.6 %)	50 (42.4 %)	
Female	117	73 (62.4 %)	44 (37.6 %)	
Age (months)				0.09^b^
mean (SD)	43.3 (12.8)	42.1 (12.0)	45.0 (13.8)	
Annual Income				<0.001^a^
< $28,000	109	83 (76.1 %)	26 (23.9 %)	
> $28,000	113	46 (40.7 %)	67 (59.3 %)	
Parental Education				<0.001^a^
< High School	60	53 (88.3)	7 (11.7)	
≥ High School	172	85 (49.4)	87 (50.6)	
Health Rating by Parent				<0.001^a^
Fair/Good	74	59 (79.7)	15 (20.3)	
Very Good	161	82 (50.9)	79 (49.1)	
Snacking Frequency				0.21^c^
Daily	224	132 (58.9)	92 (41.1)	
Other	11	9 (81.8)	2 (18.2)	
Height (cm)				0.09^b^
mean (SD)	100.1 (8.5)	99.3 (8.1)	101.3 (9.0)	
Weight (kg)				0.89^b^
mean (SD)	16.9 (3.5)	17.0 (3.3)	16.9 (3.7)	
BMI percentile				0.003^b^
mean (SD)	64.1 (30.7)	69.0 (29.2)	56.8 (31.7)	
BMI z-score				0.002^b^
mean (SD)	0.56 (1.33)	0.78 (1.26)	0.22 (1.36)	
	(median 0.64)	(median 0.89)	(median 0.31)	

^a^Chi square

^b^t test

^c^Fisher’s Exact Test

**Fig. 1:**
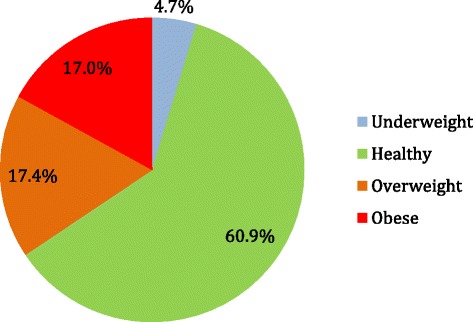
Distribution of children by BMI categories

The mean BMI z-score for the entire sample of children was above 0 (0.56 (1.33)) (median 0.64, range -4.93 to 3.06) while the mean BMI percentile was 64.1 % (30.7 %). The distribution of participating children by BMI category based upon BMI z-scores appears in Fig. 1. Overall, 17.4 % of children were classified as overweight (1.7 times the CDC expected value of 10.0 %). 17.0 % obese (3.4 times the CDC expected value of 5.0 %), and 4.7 % were underweight. However, the proportion of underweight children in the total sample approximated the expected CDC values (4.7 % vs 5.0 %).

Significantly more children with S-ECC were classified as overweight or obese than children who were caries-free (39.7 % vs. 26.6 %, *p* = 0.038). Further, analysis revealed that children with S-ECC had significantly higher mean BMI z-scores than cavity-free children (*p* = 0.002) (Table 1); ranging from -2.56 to 4.06 and -4.93 to 3.58 for the S-ECC and caries-free groupings, respectively. Similarly, children with S-ECC had significantly higher mean BMI percentiles than caries-free subjects (*p* = 0.0028); 12 % higher than the caries-free group.

BMI z-scores were found to be significantly associated with certain socioeconomic variables. As seen in Table 2, BMI z-scores and BMI percentiles were inversely associated with both income and caregiver and parental education levels. Those children who came from low income households had higher BMI z-scores and BMI percentiles (both *p* < 0.001). Similarly, children of parents and caregivers with lower levels of education also had significantly higher BMI z-scores and percentiles (*p* = 0.004 and *p* = 0.019, respectively). However, daily snacking was not associated with BMI z-scores or BMI percentiles (*p* = 0.53 and *p* = 0.70, respectively).

**Table 2:** Associations with mean BMI z-score and percentiles

Variable	N	BMI z-Score (SD)	*P*-value	BMI percentile (SD)	*P*-value
Parental Education			0.004		0.019
< High School	60	0.97 (1.31)		72.0 (28.3)	
		(median 0.96)			
≥ High School	172	0.40 (1.30)		61.2 (30.9)	
		(median 0.45)			
Annual Income			<0.001		<0.001
< $28,000	109	0.92 (1.41)		71.9 (29.7)	
		(median 1.03)			
> $28,000	113	0.19 (1.15)		56.4 (30.1)	
		(median 0.29)			
Snacking Frequency			0.53		0.70
Daily	224	0.57 (1.35)		64.3 (30.9)	
		(median 0.65)			
< Daily	11	0.31 (0.96)		60.5 (28.7)	
		(median 0.46)			
Health Rating by Parent			0.09		0.11
Very Good	161	0.77 (1.28)		68.8 (28.8)	
		(median 0.86)			
Good/Fair	74	0.46 (1.34)		61.9 (31.4)	
		(median 0.5)			

Children with optimal 25(OH)D concentrations (≥75 nmol/L) had significantly lower BMI z-scores than children with concentrations below this threshold (0.37 (1.19) vs. 0.73 (1.41), *p* = 0.04). However, no such relationship was identified between BMI percentile and vitamin D status (*p* = 0.10).

Multiple regression analyses for BMI z-score were performed (Table 3). As parental education and household income were highly associated they were not initially included together in the same regression model to avoid multicollinearity. The results of Model 1 suggest that children with S-ECC had significantly higher BMI z-scores while those from higher income households had significantly lower BMI z-scores (*p* = 0.043 and *p* = 0.004, respectively). Meanwhile, the second regression model for BMI z-scores revealed that children of parents and caregivers who completed high school or post-secondary education had lower BMI z-scores and those with S-ECC had higher z-scores (*p* = 0.029 and *p* = 0.049, respectively). A third linear regression model for BMI z-score involving backwards elimination was also performed and included both parental and caregiver education level and household income along with caries status, snacking frequency, and vitamin D status. The final iteration of this model revealed that only children with S-ECC and yearly household income were significantly and independently associated with BMI z-scores. Specifically, those with S-ECC had higher BMI z-scores (*p* = 0.045) while those from families with higher household incomes had lower scores (*p* = 0.003).

**Table 3:** Multiple linear regression for BMI z-score

Variable	Regression Coefficient (b)	Standard Error (b)	± 95 % Confidence Interval	*P* Value
Model 1				
Intercept	0.01	0.45	-	-
S-ECC (Reference = Caries-Free)	0.38	0.19	0.013, 0.75	0.043
Low Vitamin D (Reference = No)	0.19	0.18	−0.15, 0.54	0.27
Snacking Frequency (Reference = Daily)	−0.53	0.41	−0.29, 1.34	0.21
Annual Income (Reference = Low)	−0.55	0.19	−0.92, -0.18	0.004
Model 2				
Intercept	1.01	0.34	-	-
Parental Education (Reference = < High School)	−0.46	0.21	−0.87, -0.05	0.029
S-ECC (Reference = Caries-Free)	0.37	0.19	0.0009, 0.73	0.049
Low Vitamin D (Reference = No)	0.31	0.17	−0.025, 0.65	0.069
Snacking Frequency (Reference = Daily)	−0.46	0.40	−0.33, 1.25	0.26

A similar multiple linear regression analysis using backwards elimination was also undertaken for BMI percentile and included educational status, household income, snacking frequency, and caries grouping. This revealed that S-ECC and yearly household income were significantly and independently associated with BMI percentile. Those with S-ECC had higher BMI percentiles (*p* = 0.044) while whose from higher income families had lower BMI percentiles (*p* = 0.004).

## Discussion

The purpose of this study was to evaluate the relationship between BMI and S-ECC in preschool children living in southern Manitoba, Canada. Findings reveal that children with S-ECC had significantly higher BMI z-scores and corresponding BMI percentile measures than their cavity-free counterparts. According to the CDC’s BMI percentile cutoffs, significantly more children in the S-ECC group were either overweight or obese compared to caries-free children. In fact, they were 1.82 times as likely (unadjusted odds ratio) to be overweight and/or obese than their caries-free peers (39.7 % vs. 26.6 %).

Many studies involving body weight use BMI percentile as the measure of “body fatness”, as it is age and sex adjusted and allows for the ability to accurately compare children of differing ages and gender [18, 20] As stated previously, percentiles are derived from a corresponding age and sex adjusted z-score. Almost 9 % of our entire sample of children (20/235) had BMI z-scores above the corresponding 99^th^ percentile. This means that any child with a z-score > 2.34 is considered to be similar. Surprisingly though, many children had z-scores approaching and even surpassing 3.0. In light of this knowledge, the most accurate representation of the study population’s BMI comes from using z-scores.

We are not the first to study the relationship between S-ECC and BMI [14, 16, 19, 23, 24, 27, 28]. The significant association between S-ECC and higher BMI z-scores or overweight/obesity highlighted in this research is in agreement with other studies [13, 27, 35]. In contrast, other groups have reported that children with S-ECC are more likely to be underweight [14, 16, 19, 36]. Meanwhile, other large population-based studies have not found any significant association between BMI and caries experience after controlling for confounders [23, 24, 29, 37]. However, many studies in the area of BMI and caries report mean BMI, BMI percentiles, or mean weight on populations of varying ages and of mixed gender [14, 16, 28, 37–40]. The use of z-scores adjusts for both age and gender and thus allows for more meaningful reporting of means. In addition to reporting BMI z-scores in our present study, we present data on BMI percentiles to allow for comparisons with other studies in this area.

While direct causation between severe caries and obesity has not been established, the association between these two conditions is becoming apparent. Naturally, differences in inclusion criteria and case selection of children with caries and controls exist between studies, and greatly complicate our understanding of this relationship. Some studies have compared children with S-ECC with caries-free controls. However, others have compared caries-free children and those with ECC, meaning that they may have over enrolled those with milder forms of caries, thereby downplaying any potential relationships. Meanwhile, some have explored the relationship between decayed, missing and filled tooth (dmft) scores and BMI. Regardless, both caries and obesity may demonstrate associations because of shared risk factors [16, 19, 23, 27, 29]. These two chronic disease outcomes are often influenced by socioeconomic status (primarily low income and low parental education levels), overconsumption of carbohydrate-rich foods, increased frequency of snacking, and health behaviours [13, 19].

Multiple linear regression for BMI z-scores was undertaken to control for potential confounders. After controlling for snacking frequency and socioeconomic factors like low annual income and lower parental education, we identified that BMI z-scores were significantly associated with S-ECC, low annual income, and low parental education. No association was found between S-ECC and caries-free children with respect to snacking. Such a relationship may in fact exist within this population, however additional questions regarding the frequency (e.g. number of times a day), timing (e.g. *when* do the children snack?), and type (e.g. sweets, chips, fruit, vegetables, etc.) of snacking would be useful in determining whether snacking plays as crucial a role in the development of S-ECC as is commonly believed.

Overall, the high proportion of children classified as overweight or obese was rather striking. Compared to the CDC reference population, our cohort was more overweight (17.4 % vs. 10 %) and more obese (17 % vs. 5 %). 34.4 % of preschool children in our study fell into the two categories that should normally only comprise the upper 15 % of the population. A 2010 review of weight status in Manitoba children reported that 31.2 % of those 2-17 years of age were overweight or obese [41]. When restricted to 2-5 year olds, this percentage drops to 23.2 % [41]. Why, then, did our study population show such high proportions of overweight and obesity in such a young population? The answer is likely related to the study population involved, as many of the children were from families with low socioeconomic status (SES) and low parental education levels – factors which have consistently been linked with overweight and obesity. This is highlighted in the 2010 review by Yu, et al., who found that 40.8 % of the low-income children qualified as overweight or obese [41].

A unique finding of this study was that children with optimal vitamin D status, as measured by 25(OH)D concentrations, had significantly lower BMI z-scores than children with levels below this threshold. This seems to coincide with the observations from a study on the vitamin D status of First Nations children from two Cree communities that increased BMI may be associated with lower vitamin D concentrations [42]. This could possibly be due to poor quality, but high calorie diets.

Naturally, this study had certain limitations. As the present investigation relied on data which was cross-sectional in nature, we were unable to establish a cause and effect relationship between the variables. Recall bias may have played a role in certain questions within the questionnaires. While the S-ECC and caries-free groups were relatively matched for age and sex, there are some inherent differences between the groups that were impossible to control, which we discussed in two previous papers involving this cohort of children [9, 10]. For instance, one cannot address factors like the household income and parental education as they are key determinants for S-ECC [9, 10]. However, education and income were included in the regression models to control for their effects. Another limitation was that weight and height measurements for those undergoing dental surgery were obtained from the chart though the measurements had been performed by a trained healthcare professional and those recruited from the community were obtained by a trained study staff. However, all used calibrated scales and stadiometers. Unfortunately, the degree of intra and inter-rater reliability with respect to height and weight measurements was not determined. The results from this study are not necessarily generalizable to the general population outside of Manitoba.

The identified association between BMI z-score and S-ECC is important for both the dental and medical professions to appreciate. Dentists should consider screening their pediatric patients with caries for obesity and refer at-risk children to a physician or other primary health care provider for follow-up. Dentists and dental hygienists traditionally deliver dietary counselling as part of their preventive approach to minimize the risk of caries. However, the benefits of this type of dietary counselling to avoid sugars and junk foods may also extend to the child’s general health and may help lower the child’s risk for obesity [29, 43]. Members of the dental team are well positioned to be allies in the battle against childhood obesity. Medical professionals caring for children who are at-risk for being overweight and obese should understand that these children may also have serious dental needs. The dietary and lifestyle counselling that they provide can have positive impacts on a child’s oral health.

## Conclusions

Overall, children in this sample were 1.7 times more likely to be overweight and 3.4 times more likely to be obese when compared to the CDC’s expected BMI percentiles. Children with S-ECC were significantly more likely to be overweight and obese compared to caries-free children. Children with S-ECC in our sample had significantly higher BMI z-scores and percentiles than caries-free peers. Members of the dental team are well positioned to be allies in the battle against childhood obesity.

## Abbreviations

AAPD: American academy of pediatric dentistry
BMI: Body mass index
CDC: Center for disease control
dmft: decayed, missing, filled teeth score
ECC: Early childhood caries
GA: General anesthesia
MHC: Misericordia health centre
SD: Standard deviation
S-ECC: Severe early childhood caries
SES: Socioeconomic status
